# Sequelae of preterm birth over the lifespan: an exploratory analysis of behavioral problems in childhood and increased risk of major depression and anxiety in adulthood from a cohort study

**DOI:** 10.1016/j.eclinm.2025.103316

**Published:** 2025-07-08

**Authors:** Achim Fieß, Alica Hartmann, Mareike Ernst, Alexander K. Schuster, Eva Mildenberger, Elmar Brähler, Michael S. Urschitz, Norbert Pfeiffer, Manfred E. Beutel, Sandra Gißler, Jonas Tesarz

**Affiliations:** a Department of Ophthalmology, University Medical Center of the Johannes Gutenberg University Mainz, Mainz, Germany; b Department of Psychosomatic Medicine and Psychotherapy, University Medical Center of the Johannes Gutenberg University Mainz, Mainz, Germany; c Department of Clinical Psychology, Psychotherapy and Psychoanalysis, Institute of Psychology, University of Klagenfurt, Klagenfurt am Wörthersee, Austria; d Division of Neonatology, Department of Pediatrics, University Medical Center of the Johannes Gutenberg-University Mainz, Germany; e Department of Medical Psychology and Medical Sociology, University of Leipzig Medical Center, Germany; f Division of Paediatric Epidemiology, Institute of Medical Biostatistics, Epidemiology, and Informatics, University Medical Centre of the Johannes Gutenberg University Mainz, Germany; g Department of General Internal Medicine and Psychosomatics, University Hospital Heidelberg, Heidelberg University, Heidelberg, Germany

**Keywords:** Prematurity, Fetal growth restriction, Mental health, Adult outcomes, Maternal risk factors

## Abstract

**Background:**

Existing research has shown that prematurity and low birth weight are associated with mental health in children and adolescents, but their impact on mental health in adulthood and the mechanisms involved have been less studied. This study investigated the impact of prematurity and abnormal fetal growth, as a marker for fetal growth restriction, on mental health outcomes in adulthood.

**Methods:**

This retrospective cohort study examined adults aged 18–52 years, categorized by gestational age (extremely preterm: ≤28 weeks; very preterm: 29–32 weeks; moderately preterm: 33–36 weeks; term: ≥37 weeks). The cohort consists of individuals who were either born preterm or at term from 1969 to 2002. Data collection occurred between 2019 and 2021. This exploratory analysis used multivariable logistic regression to assess the impact of prematurity and fetal growth restriction on adult mental health outcomes (measured by the “Patient Health Questionnaire”), adjusting for age, gender, and socioeconomic status. The study also explored the relationship between childhood behavioral problems, retrospectively described by the mothers of the participants (2–4 years), and adult mental health, with separate analyses for preterm and term births.

**Findings:**

The multivariable logistic regression analysis of 606 participants (average age 28·96 ± 8·9 years, 326 females) indicated that a low gestational age of ≤28 weeks was independently associated with an increased prevalence of major depressive disorder (OR = 4·14, CI: 1·43–11·77, p = 0·01) and anxiety disorder (OR = 5·17, CI: 1·51–17·37, p = 0·01) in adulthood compared to a gestational age of ≥37 weeks. A low birth weight percentile was not associated with mental disorders. Further regression analysis revealed that peer problems and emotional problems in childhood were significantly associated with major depression in adulthood in participants born preterm. The sensitivity analysis revealed that the previously observed association between the gestational age group of ≤28 weeks and major depressive disorder was no longer statistically significant (OR = 2·84, CI: 0·85–9·07, p = 0·08) when the model was adjusted for maternal smoking and alcohol consumption during pregnancy. The association between the extreme preterm group and generalized anxiety disorder remained significant (OR = 4·63, CI: 1·27–16·22, p = 0·02).

**Interpretation:**

This study provides exploratory insights into the complex impact of prematurity on mental health in adulthood, highlighting the vulnerability of the extremely preterm group (≤28 weeks) from a multi-informant, lifespan perspective. It emphasizes the importance of continuous monitoring and psychosocial support for those born extremely preterm, from infancy through adulthood, to mitigate potential adverse mental health outcomes.

**Funding:**

The Gutenberg Prematurity Study was supported by the 10.13039/501100008436Ernst und Berta-Grimmke Stiftung, Stufe 1 support of the UM and the 10.13039/501100003042Else Kröner-Fresenius-Stiftung.

**Research in context**

**Evidence before this study**

Research links prematurity or low birth weight to mental health issues in youth, but studies in adults rarely separate the effects of prematurity and low birth weight or consider their specific degrees. Further, the mechanisms through which early developmental challenges influence mental health outcomes later in life remained under-explored, particularly the role of childhood behavioral issues. This study aimed to fill these gaps by examining the long-term effects of prematurity and abnormal fetal growth on adult mental health outcomes, providing a comprehensive view from a multi-informant, lifespan perspective.

**Added value of this study**

In this retrospective cohort study, which included a prospective examination of 606 participants, we observed that a gestational age of ≤28 weeks significantly increased the risk of major depressive disorder (OR = 4·14, CI: 1·43–11·77) and anxiety disorder (OR = 5·17, CI: 1·51–17·37) in adulthood compared to ≥37 weeks. The birth weight percentile was not associated with negative mental health outcomes in adulthood. Childhood peer and emotional problems, reported by mothers, were strongly associated with adult major depression in preterm individuals. The sensitivity analysis revealed that the previously observed association between the gestational age group of ≤28 weeks and major depressive disorder was no longer statistically significant (OR = 2·84, CI: 0·85–9·07, p = 0·08) when the model was adjusted for maternal smoking and alcohol consumption during pregnancy. The association between the extreme preterm group and generalized anxiety disorder remained significant (OR = 4·63, CI: 1·27–16·22, p = 0·02).

**Implications of all the available evidence**

These findings suggest a significant impact of prematurity but not fetal growth restriction on mental health in adulthood, highlighting the importance of early emotional and social development in mitigating long-term mental health risks.

## Introduction

Prematurity and fetal growth restriction are distinct conditions that present a significant global challenge due to their profound impact on morbidity, mortality, and socioeconomic outcomes.^1^, ^2^, ^3^ Prematurity refers to infants born before completing 37 weeks of gestation,^4^ while fetal growth restriction can manifest as fetuses being small for gestational age (SGA), referring to infants who weigh less than average for their gestational age irrespective of the cause.^5^ The causes and consequences of prematurity and fetal growth restriction may differ, yet both are regarded as risk factors for the subsequent impaired physical and psychological development.

In particular, prematurity is the leading cause of neonatal mortality worldwide, affecting approximately 13·4 million babies in 2020 and representing around 9·9% of all births.^6^ Despite the increasing prevalence of preterm births,^7^ advances in medical care have improved the survival rates of these infants, allowing more of them to reach adulthood. However, the health consequences of prematurity and fetal growth restriction often persist throughout life, affecting both physical and psychological dimensions. Previous studies have shown that premature birth or low birth weight increased the risk of mental disorders in childhood and adolescence,^8^, ^9^, ^10^ highlighting the necessity for continued research into the long-term mental health outcomes of those born preterm.

Mood disorders are particularly prevalent, with depression and anxiety disorders being the most common.^11^^,^^12^ Depression is a widespread and debilitating mental disorder, with meta-analyses estimating it affects approximately 2·8% of children under 13 years,^13^ 5·6% of adolescents aged 13–18 years,^13^ and 12·9% of adults.^14^ Additionally, it has been increasing in prevalence in recent years.^15^ Moreover, depression is a significant risk factor for suicide,^16^ highlighting the urgent need to identify and comprehend its underlying causes. Given the well-documented physical and psychological consequences of prematurity, research into the potential association between prematurity and fetal growth restriction with the development of depression in later life is of particular importance. A Finnish study of 37,682 cases and 148,795 matched controls found an association between premature birth (below 28 weeks gestation) and childhood depression.^8^ A study of 2032 adolescents investigating the association between prematurity (3 weeks prior to term)/low birth weight (<2500 g) and depressive disorder found an 11-fold increased odds for depressive disorder.^17^ However, it is still unclear to what extent preterm birth also affects mental health in adulthood.

Burnett and colleagues assessed 297 extremely preterm/low birth weight (<1000 g) individuals and 260 controls with normal birth weight from 1991 to 1992 showing similar mental health outcomes at the age of 25 but the study did not distinguish between different levels of prematurity and perinatal hypotrophy.^18^ There is also an increased prevalence of anxiety symptoms in childhood due to prematurity,^19^ with more internalizing problems in preterm individuals, such as anxiety.^20^ Furthermore, a higher prevalence of addictions^21^ or eating disorders in individuals born preterm was observed.^22^ A Danish study found that male individuals born preterm or growth-restricted were more susceptible to alcoholism,^21^ while other studies report reduced alcohol consumption in individuals born preterm.^23^ There are also contradictory results regarding the association with eating disorders with some studies reporting an association with preterm birth, while other studies could not confirm this association.^24^ In summary, while evidence suggests that prematurity and fetal growth restriction influence mental health outcomes in childhood and adolescence, their long-term effects on adult mental health remain insufficiently explored. Understanding these impacts requires consideration of additional factors, such as maternal behaviors during pregnancy. Maternal smoking and alcohol consumption, known contributors to preterm birth and fetal growth restriction, are well-established risk factors for adverse neonatal and mental health outcomes.

Maternal smoking during pregnancy has been extensively studied as an independent risk factor for preterm birth and fetal growth restriction, with studies consistently demonstrating its adverse effects on neonatal outcomes.^25^, ^26^, ^27^ Prenatal alcohol exposure has similarly been linked to increased risks of preterm birth and fetal growth restriction.^28^^,^^29^ Beyond these effects, maternal smoking and alcohol consumption during pregnancy are well-established predictors of adverse mental health outcomes in offspring. For instance, maternal smoking during pregnancy has been associated with an increased risk of depression, anxiety, and behavioral problems in children and adults.^30^ Similarly, prenatal alcohol exposure has been linked to heightened vulnerability to mental health disorders, including anxiety, depression, and substance use disorders.^31^ These findings underscore the need to account for prenatal exposures, such as maternal smoking and alcohol consumption, when examining the developmental pathways shaping mental health outcomes.

In addition to these prenatal influences, research increasingly highlights the role of early emotional and behavioral problems in preterm infants as key contributors to mental health risks later in life. Emotional and behavioral difficulties observed in early childhood (ages 2–4 years) often persist into adolescence and adulthood, contributing to an elevated risk of developing depression and anxiety disorders. Linsell et al.^32^ demonstrated that preterm infants experience higher rates of emotional and behavioral problems across childhood, adolescence, and into early adulthood. Similarly, Eriksen et al.^33^ found that early behavioral issues predict heightened emotional problems in adulthood, particularly among preterm individuals.

This study explores whether emotional and behavioral problems in early childhood (ages 2–4) are associated with an increased risk of mental health disorders in later life. By examining early-life emotional and behavioral influences, the study seeks to shed light on the elevated prevalence of mental health issues among individuals born preterm and offer insights into potential developmental pathways that impact adult mental health. Theoretical frameworks, such as the Developmental Origins of Health and Disease (DOHaD) model,^34^^,^^35^ suggest that early life stressors, including preterm birth, can disrupt neurodevelopment, creating long-term vulnerability to mental disorders. Therefore, this study analyzed the associations between gestational age and birth weight percentile, as surrogate markers for fetal growth restriction, with mental health outcomes in later life. These outcomes are assessed using the psychological dimensions of the Patient Health Questionnaire (PHQ-D)^36^ including somatoform syndrome, major depressive disorder, other depressive syndromes, anxiety disorder, and alcohol-related disorders. By identifying whether associations previously observed in childhood persist into adulthood, this research aims to bridge a significant knowledge gap and enhance our understanding of long-term mental health trajectories. Specifically, we aimed to model the developmental pathways from birth through adulthood by examining how gestational age, birth weight percentile, maternal smoking, and prenatal alcohol exposure influence mental health outcomes from birth to adulthood (ages 18–52 years). These factors were included based on robust evidence linking them to both prematurity and long-term mental health risks. The PHQ-D allows for precise differentiation between depressive and anxiety disorders, a distinction that previous studies have not always made.

## Methods

### Study population

The Gutenberg Prematurity Study (GPS) is a retrospective cohort study performed at the University Medical Center of the Johannes Gutenberg University Mainz (UMCM) in Germany. The GPS includes individuals who were either born preterm or at term from 1969 to 2002, aged between 18 and 52 years on study entry. The research approach merged elements of a retrospective cohort with the prospective collection of data. Every second individual born preterm at 33–36 weeks and all those born at 32 weeks or less at UMCM from 1969 to 2002 were invited to participate in this study. A matched control group by age and sex was created by recruiting six term individuals (three males, three females) within the 10th–90th birth weight percentiles for each birth month from 1969 to 2002.^37^ To stratify for perinatal fetal nutrition effects independent of prematurity, 40 term participants were included with birth weights: below the 3rd percentile (severely SGA), 3rd–<10th percentile (moderately SGA), >90th–97th percentile (moderately large for gestational age), and above the 97th percentile (severely large for gestational age), matched by age and sex. Inclusion and exclusion criteria are detailed in Supplemental Figure S1.

The participants were grouped according to their gestational age: Group 1 was composed of individuals born at a gestational age of 37 + 0 weeks or more (n = 296); Group 2 contained those born between 33 + 0 and 36 + 6 weeks of gestation (n = 137); Group 3 included those born between 29 + 0 and 32 + 6 weeks (n = 116); Group 4 consisted of those born at or before 28 + 6 weeks of gestation (n = 57).

To further enrich the dataset, mothers of participants were invited to complete a basic demographic questionnaire and the Strengths and Difficulties Questionnaire (SDQ), a standardized behavioral assessment tool for children aged 2–4 years. A total of 217 mothers participated, with the following group distribution: Group 1: 112 mothers; Group 2: 49 mothers; Group 3: 33 mothers; and Group 4: 23 mothers. Data collection from both participants and their mothers occurred between 2019 and 2021.

### Ethics

All participants and their participating mothers provided written informed consent in accordance with Good Clinical Practice, Good Epidemiological Practice, and the Declaration of Helsinki. The study protocol was approved by the Medical Chamber of Rhineland-Palatinate's ethics committee (reference no. 2019-14161; original vote: 29.05.2019, latest update: 02.04.2020). Additional information regarding the study methodology and reported results are available elsewhere.^38^, ^39^, ^40^

### Data collection

The adult participants underwent comprehensive medical assessments conducted by trained healthcare professionals between 2019 and 2021, which included evaluations of physical health, such as measurements of height, weight, Body Mass Index, waist-to-hip ratio, and vital signs like blood pressure and heart rate. Blood samples were also collected to determine relevant laboratory parameters. The participants were first subjected to detailed sociodemographic and clinical phenotyping via a questionnaire and then invited to a clinical interview with a physical examination during which the questionnaire data were validated and missing information was added. Socioeconomic status (SES) was assessed using the Robert Koch Institute's Federal Health Multidimensional Index for the National Health Survey in Germany^41^ and the three subdimensions, education, occupation, and income, were transformed into a sum score (3–21).^41^ Mental health status, along with medical history, lifestyle factors, and psychosocial variables, was assessed through structured personal interviews conducted by qualified clinical interviewers, with responses documented in written form. To enhance the accuracy of neonatal information, birth records were retrieved and cross-referenced with participants' reports, ensuring reliable early-life health data. Additionally, all participants were encouraged to bring current medical records to allow for cross-referencing with health information from adulthood. Participant recruitment and assessments were paused for approximately eight weeks during the early phase of the COVID-19 pandemic in 2020. The responsible hygiene department evaluated our study procedures, and after implementing appropriate protective measures (e.g., mask-wearing, enhanced hygiene protocols), assessments were resumed. These measures were intended to ensure participant and staff safety without compromising the quality of the collected data.

Mothers of the adult participants were invited to take part in the study alongside their children. They were asked to attend in-person assessments at the study center, where they completed a self-administered questionnaire and participated in a structured clinical interview conducted by trained interviewers. The questionnaire included the SDQ to retrospectively assess their child's emotional and behavioral problems during early childhood (ages 2–4 years). Additionally, information on prenatal alcohol consumption and maternal smoking during pregnancy was obtained through self-reported data collected in the same questionnaire. These assessments were designed to capture early-life risk factors relevant to the mental health outcomes of the offspring.

### Assessment of pre- and postnatal history

Data regarding gestational age (in weeks), birth weight (in kilograms), placental insufficiency, maternal smoking, preeclampsia, and breastfeeding were retrieved from the participants' medical records archived at the UMCM. Birth weight percentiles were calculated according to the methodology outlined by Voigt et al.^42^ These percentiles are derived from a large dataset of singleton births in Germany, covering gestational ages from 20 to 42 weeks, and include detailed percentile values for each week of gestation. This approach enables accurate classification of fetal growth restriction and large-for-gestational-age (LGA) infants, adjusted for gestational age, thus minimizing potential confounding between birth weight and prematurity. By using these gestational age-adjusted percentiles rather than absolute birth weights, we account for the expected variations in birth weight across different gestational ages and enhance the precision of growth assessments in preterm as well as term infants.

### Emotional and behavioral problems in early childhood (maternal reports)

Emotional and behavioral problems during early childhood were assessed using the Strengths and Difficulties Questionnaire (SDQ),^43^^,^^44^ a behavioral questionnaire that assesses strengths and difficulties and is available for ages 2–4 years. The questionnaire was completed retrospectively by the participants' mothers and assessed five dimensions: conduct problems, emotional problems, hyperactivity score, peer problems, and prosocial score. Each subscale contained five items scored from 0 to 2, thus each subscale ranged from 0 to 10. The total difficulties score, which excludes the prosocial score, ranged from 0 to 40 and was calculated by summing the scores from the first four dimensions. The SDQ demonstrated an overall Cronbach's α of 0·76 (CI: 0·72–0·78) indicating good reliability.^45^ We chose the standardized SDQ over self-reported mental health issues to ensure objectivity, comparability, and comprehensiveness in our assessment of emotional and behavioral difficulties. While self-reported diagnoses are subject to recall bias and may overlook undiagnosed or untreated conditions, the SDQ provides a validated, systematic approach that captures a broader spectrum of mental health issues, including subclinical difficulties.

### Mental health outcomes in adulthood

Mental health outcomes in adulthood were assessed using the German version of the Patient Health Questionnaire (PHQ-D).^36^ This tool, developed for screening mental disorders, encompasses seven mental health conditions: somatoform syndrome, major depressive disorder, other depressive symptoms, panic syndrome, binge eating, bulimia nervosa, and alcohol-related disorders. Generalized anxiety disorder was assessed using the Generalized Anxiety Disorder 7-item scale (GAD-7).^46^ The PHQ-D and GAD-7 are widely used in epidemiological research due to their strong validity and reliability in identifying these mental health disorders.^47^, ^48^, ^49^ Representative population studies have also confirmed the good psychometric properties of its dimensions.^50^^,^^51^ The scoring methods and cut-off values for determining these disorders can be found in the PHQ-D manual.^48^ It is important to note that the PHQ-D and its modules assess mental health outcomes based on different time frames. For instance, depressive symptoms are measured over the past two weeks, while anxiety symptoms (GAD-7) are assessed based on a four-week period.

### Exposures

The exposures considered as potential factors affecting the main outcome measures were gestational age categories (≤28 weeks, 29–32 weeks, and 33–36 weeks) and birth weight percentile.

### Statistical analysis

The absolute and relative frequencies were determined for dichotomous parameters, with the mean and standard deviation calculated for normally distributed variables. Separate univariable logistic regressions were conducted for the preterm and term birth groups to assess the impact of early childhood emotional and behavioral problems on mental health outcomes in adulthood. In each group, the association between early childhood issues and adult mental health outcomes was examined. These analyses were adjusted for age, gender, and socioeconomic status to control for their potential influence on mental health. This approach enables a comparison of the effects of early emotional and behavioral problems on adult mental health between individuals born preterm and those born at term. Multivariable logistic regression analyzed mental health dimensions from the PHQ-D as primary outcome, namely: somatoform syndrome, major depressive disorder, other depressive syndromes, anxiety disorder, and alcohol-related disorders. Bulimia nervosa and binge-eating were excluded due to insufficient cases (Bulimia: 5; Binge-eating: 17). Independent variables included gestational age categories (33–36 weeks, 29–32 weeks, and ≤28 weeks) and birth weight percentile. Gestational age and birth weight percentile were analyzed as continuous variables adjusting for gender, age, and socioeconomic status to explore dose-dependent effects. A sensitivity analysis was performed by integrating the variables maternal alcohol consumption during pregnancy and maternal smoking during pregnancy into the model. This analysis was conducted to evaluate whether the inclusion of these factors alters the primary associations observed in the main analysis. Additionally, a further sensitivity analysis was carried out to assess the impact of the COVID-19 pandemic on our study, including a variable indicating whether participants were assessed before or after the pandemic began.

This is an explorative study so no adjustment for multiple testing was performed. R version 4·3·2 (R Core Team) was used for the statistical evaluation.^52^

### Role of funding source

The Gutenberg Prematurity Study was supported by the Ernst und Berta-Grimmke Stiftung, Stufe 1 support of the UM and the Else Kröner-Fresenius-Stiftung. The funders had no role in study design, data collection and analysis, decision to publish, or preparation of the manuscript.

## Results

### Participant characteristics

The sample was comprised of 606 individuals (age 28·96 ± 8·9 years, 326 women) born preterm or term, and their descriptive characteristics and perinatal parameters are shown in Table 1. The earlier gestational age at birth, the higher the frequency of intubation and the longer the duration of both incubator use and neonatal intensive care unit (NICU) stay during the neonatal period. In adulthood, the prevalence of health conditions across the groups was generally low, likely due to the relatively young age of participants. However, arterial hypertension was more frequently observed across all groups, with rates of 28·0% in the term group (GA ≥ 37), 29·2% in the GA 33–36 group, 29·3% in the GA 29–32 group, and 26·3% in the extremely preterm group (GA ≤ 28). Epilepsy showed a markedly higher prevalence in the ≤28 weeks gestational age group compared to the other groups. Of the total sample (N = 606), maternal reports on early childhood emotional and behavioral problems using the SDQ were available for 217 participants (35·8%). The mothers had an average age of 59·27 ± 6·93 years. Regarding prenatal risk factors, maternal smoking during pregnancy was reported by 12 mothers (4·1%) in the GA ≥ 37 weeks groups and by 8 mothers (14·0%) in the extremely preterm group. Similarly, maternal alcohol consumption during pregnancy was reported by 6 mothers (2·0%) in the term group and by 5 mothers (8·8%) in the extremely preterm group. Additionally, seeking professional psychological help during pregnancy or after birth was reported by 7 mothers (6·3%) in the term group and by 3 mothers (13·0%) in the extremely preterm group.

**Table 1:** Demographic and neonatal characteristics of adult offspring (n = 606) and maternal characteristics (n = 217) by study group in the Gutenberg Prematurity Study.

	Group 1	Group 2	Group 3	Group 4	p-value
	GA ≥ 37	GA 33–36	GA 29–32	GA ≤ 28	
**Number of participants (n)**	**296**	**137**	**116**	**57**	
Age (y), *M* ± SD	30·01 ± 9·4	29·47 ± 9·2	27·88 ± 8·0	24·51 ± 5·5	<0·001
Female participants (%)	156 (52·7%)	82 (59·9%)	60 (51·7%)	28 (49·1%)	0·41
Socioeconomic status (SES)					<0·001
Low SES (<9), n (%)	34 (11·5%)	24 (17·5%)	17 (14·7%)	16 (28·1%)	
Moderate SES (9–15), n (%)	171 (57·8%)	81 (59·1%)	82 (70·7%)	39 (68·4%)	
High SES (16–21), n (%)	91 (30·7%)	32 (23·4%)	17 (14·7%)	2 (3·5%)	
Birth weight, *M* ± SD	3436 ± 900	2068 ± 464	1500 ± 364	855 ± 215	<0·001
BW < 1500 g (yes), n (%)	1 (0·3%)	13 (9·5%)	55 (47·4%)	57 (100%)	<0·001
BW < 1000 g (yes), n (%)	0 (0·0%)	0 (0·0%)	10 (8·6%)	41 (71·9%)	<0·001
Birth weight percentile, *M* ± SD	49·48 ± 36·9	25·25 ± 24·2	43·09 ± 26·4	37·60 ± 24·8	<0·001
Birth weight percentile groups					<0·001
Severe SGA <3 percentile	40 (13·5%)	20 (14·6%)	4 (3·4%)	1 (1·8%)	
Moderate SGA 3–<10 percentile	38 (12·8%)	26 (19·0%)	6 (5·2%)	9 (15·8%)	
Moderate LGA >90–97 percentile	38 (12·8%)	1 (0·7%)	2 (1·7%)	0 (0%)	
Severe LGA > 97 percentile	40 (13·5%)	2 (1·5%)	0 (0%)	0 (0%)	
Multiple birth (yes)	18 (6·1%)	55 (40·1%)	37 (31·9%)	13 (22·8%)	<0·001
Gestational age (weeks), *M* ± SD	39·22 ± 1·5	34·31 ± 1·0	30·47 ± 1·2	26·32 ± 1·4	<0·001
Range (min-max)	(37–43)	(33–36)	(29–32)	(23–28)	
Intubation (yes), n (%)	3 (1·0%)	17 (12·4%)	67 (57·8%)	52 (91·2%)	<0·001
Stay in incubator (days), *M* ± SD	1·27 ± 4·9	8·72 ± 10·8	25·47 ± 16·6	58·99 ± 22·9	<0·001
Stay in intensive care unit (days), *M* ± SD	0·41 ± 2·0	5·30 ± 13·3	25·62 ± 25·3	72·71 ± 32·3	<0·001
Medical history in adulthood					
Arterial Hypertension^a^, n (%)	83 (28·0%)	40 (29·2%)	34 (29·3%)	15 (26·3%)	0·94
Type 1 diabetes, n (%)	3 (1·0%)	1 (0·7%)	1 (0·9%)	0 (0·0%)	0·89
Type 2 diabetes, n (%)	3 (1·0%)	2 (1·5%)	0 (0·0%)	0 (0·0%)	0·52
Hyperlipidemia, n (%)	8 (2·7%)	2 (1·5%)	1 (0·9%)	0 (0·0%)	0·38
Myocardial infarction, n (%)	1 (0·3%)	0 (0·0%)	0 (0·0%)	0 (0·0%)	0·79
Autoimmune disease, n (%)	1 (0·3%)	2 (1·5%)	2 (1·7%)	2 (3·5%)	0·18
Epilepsy, n (%)	1 (0·3%)	3 (2·2%)	9 (7·8%)	8 (14·0%)	<0·001
Preeclampsia (yes), n (%)	31 (10·5%)	24 (17·5%)	16 (13·8%)	11 (19·3%)	0·12
Placental insufficiency (yes), n (%)	13 (4·4%)	16 (11·7%)	3 (2·6%)	3 (5·3%)	0·01
HELLP syndrome (yes), n (%)	1 (0·3%)	6 (4·4%)	2 (1·7%)	3 (5·3%)	0·01
Gestational diabetes (yes), n (%)	5 (1·7%)	7 (5·1%)	2 (1·7%)	1 (1·8%)	0·17
Breastfeeding (yes), n (%)	170 (57·4%)	75 (54·7%)	59 (50·9%)	25 (43·9%)	0·24
**Number of maternal participants**	**112**	**49**	**33**	**23**	
Age (y), *M* ± SD	60·41 ± 7·48	57·84 ± 6·52	58·34 ± 5·82	58·04 ± 5·72	0·09
Maternal smoking during pregnancy (yes), n (%)	12 (4·1%)	4 (3·0%)	9 (7·8%)	8 (14·0%)	0·77
Maternal alcohol consumption during pregnancy (yes), n (%)	6 (2·0%)	2 (1·5%)	3 (2·6%)	5 (8·8%)	0·32
Seeking psychological help during pregnancy or after birth (yes), n (%)	7 (6·3%)	5 (10·2%)	5 (15·2%)	3 (13·0%)	0·39

BW–birth weight; GA–gestational age; SES–socioeconomic status; *M*−mean; SGA–small for gestational age; LGA–large for gestational age; SD–standard deviation; y–year.

a Arterial hypertension was defined by antihypertensive medication use, systolic blood pressure >140 mmHg, diastolic blood pressure >90 mmHg, or an established diagnosis.

Furthermore, descriptive variations in mental health outcomes in adulthood were found among the study groups (Table 2), with major depressive disorder being most common in the ≤28 weeks group (16·3%), followed by anxiety disorder (12·2%).

**Table 2:** Maternal-reported emotional and behavioral problems in childhood (ages 2–4) and self-reported mental health outcomes in adulthood (ages 18–52 years).

	Group 1	Group 2	Group 3	Group 4	p-value^a^
	GA ≥ 37	GA 33–36	GA 29–32	GA ≤ 28	
Number of participants	296	137	116	57	
**Emotional and behavioral problems in childhood (SDQ)**					
Conduct problems, *M* (SD)	1·44 (1·15)	1·20 (1·01)	1·22 (1·23)	1·64 (1·52)	0·31
Emotional problems, *M* (SD)	1·22 (1·62)	1·89 (2·00)	2·17 (2·05)	2·96 (2·13)	<0.001
Hyperactivity score, *M* (SD)	1·91 (1·98)	2·48 (2·09)	2·88 (2·38)	4·54 (2·38)	<0.001
Peer problems, *M* (SD)	1·09 (1·38)	1·04 (1·06)	1·98 (2·27)	3·62 (2·67)	<0·001
Prosocial score, *M* (SD)	8·21 (1·76)	8·32 (1·78)	8·12 (1·87)	7·28 (2·59)	0·12
Total difficulties score, *M* (SD)	5·79 (4·22)	6·14 (3·24)	8·06 (5·03)	12·96 (5·83)	<0.001
**Mental health outcomes in adulthood (PHQ-D)**					
Somatoform syndrome (yes), n (%)	21 (7·1%)	6 (4·5%)	7 (6·5%)	5 (10·0%)	0·58
Major depressive disorder (yes), n (%)	10 (3·4%)	4 (3·0%)	3 (2·8%)	8 (16·3%)	<0·001
Other depressive symptoms (yes), n (%)	36 (12·2%)	22 (16·7%)	16 (15·0%)	11 (22·4%)	0·24
Generalized anxiety disorder (yes), n (%)	7 (2·4%)	3 (2·3%)	5 (4·7%)	6 (12·2%)	0·005
Bulimia nervosa (yes), n (%)	3 (1·0%)	1 (0·8%)	0 (0·0%)	1 (1·9%)	0·63
Binge-eating (yes), n (%)	10 (3·4%)	4 (3·0%)	2 (1·9%)	1 (1·9%)	0·84
Alcohol-related disorders (yes), n (%)	29 (9·8%)	7 (5·3%)	6 (5·6%)	1 (1·9%)	0·10

Emotional and behavioral problems were assessed retrospectively via the SDQ at age 2–4 and mental health outcomes in adulthood were assessed via the PHQ-D and GAD-7.

a p-values were calculated using one-way analysis of variance (ANOVA) for continuous variables and chi-squared tests for categorical variables.

### Uni- and multivariable regression analysis

**Fig. 1:**
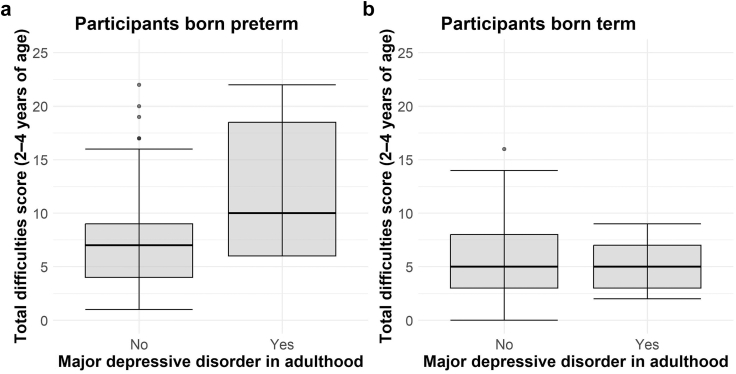
**Total Strength and Difficulties scores (SDQ, range 0–40) in childhood (2–4 years of age) stratified by the presence of major depressive disorder in adulthood in participants born a) preterm and b) term. Higher values indicate more emotional and behavioral problems during early childhood**.

**Fig. 2:**
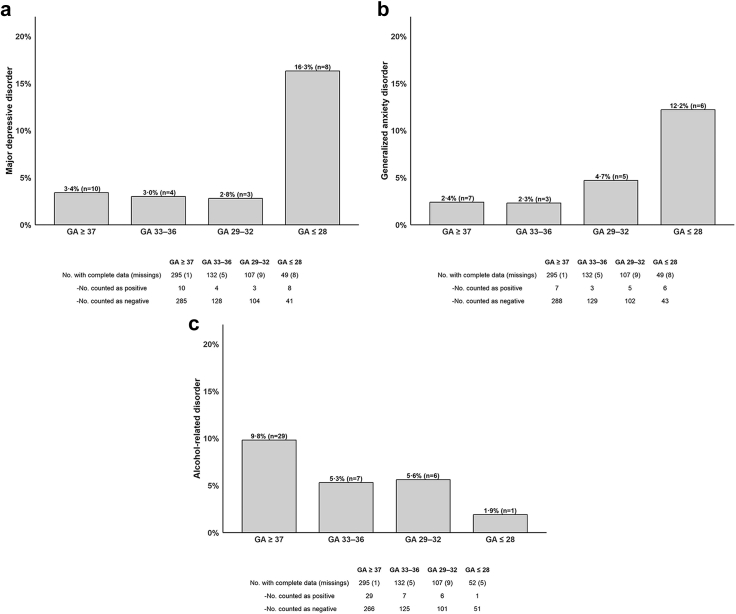
**Prevalence of a) major depressive disorder, b) generalized anxiety disorder, and c) alcohol-related disorders stratified by gestational age**.

Regarding the associations between early childhood (2–4 years) emotional and behavioral problems reported by mothers and adult mental disorders, which were analyzed in a smaller subsample of 217 participants, early peer problems (OR = 1·71, CI: 1·26–2·44, p = 0·001) and emotional problems (OR = 1·42, CI: 1·00–2·05, p = 0·05) were linked to major depressive disorder in adulthood but only in the preterm group (<37 weeks gestation). No such associations were found in the term group (≥37 weeks gestation) (Supplemental Table S4, Fig. 1). Individuals born with a gestational age of ≤28 weeks were at higher risk of major depressive disorder and generalized anxiety disorder in adulthood (Table 3, Fig. 2). These associations remained relevant after adding the birth weight percentile to the analysis which was not associated with any of the mental outcomes. Model 1 showed fewer alcohol-related disorders with a gestational age of ≤28 weeks (OR = 0·13, CI: 0·01–0·64, p = 0·05), but this association was not significant in Model 2 which included birth weight percentile. When considering SES, higher SES was found to be significantly associated with a lower risk of experiencing a somatoform syndrome (OR = 0·87, CI: 0·78–0·97, p = 0·01) and major depressive disorder (OR = 0·84, CI: 0·73–0·96, p = 0·01) in adulthood. With respect to gender, women were found to have a significantly higher risk of somatoform syndrome (OR = 5·88, CI: 2·55–16·07, p < 0.001), other depressive symptoms (OR = 1·79, CI: 1·11–2·96, p = 0·02) and generalized anxiety disorder (OR = 3·89, CI: 1·39–13·91, p = 0·02) compared to males. In contrast, males had an increased likelihood of alcohol-related disorders (OR = 0·23, CI: 0·10–0·46, p < 0·001). Analysis with continuous parameters revealed that the risk of generalized anxiety disorder increased by 11·4% per week decrease in gestational age, depression risk increased by 10% per week decrease, and alcohol-related disorder risk increased by 11% per additional week of gestational age (Supplemental Table S1). The first sensitivity analysis revealed that the previously observed association between the gestational age group of ≤28 weeks and major depressive disorder was no longer statistically significant (OR = 2·84, CI: 0·85–9·07, p = 0·08) when including maternal smoking and alcohol consumption during pregnancy in the model (Supplemental Table S2). A significant association was shown between maternal alcohol consumption during pregnancy and the occurrence of major depressive disorder in offspring in adulthood (OR = 18·60, CI: 5·24–65·76, p < 0·001). However, these findings should be interpreted with caution due to the small number of mothers reporting alcohol consumption (n = 16) and smoking (n = 33) during pregnancy, which limits the statistical power of this analysis. The association between gestational age and anxiety disorders remained consistent. Additionally, an association between GA ≤ 28 weeks and the occurrence of other depressive symptoms was identified, which was not apparent in the primary analysis. A second sensitivity analysis examining the impact of COVID-19 pandemic on our study showed no association between mental disorders and whether participants were surveyed before or after begin of the pandemic (Supplemental Table S3). The association between GA ≤ 28 weeks and major depressive disorder persisted. The previously significant correlation with generalized anxiety disorder narrowly missed significance (OR = 3·52, CI: 0·94, 13·28, p = 0·06). No correlation with the pandemic period was observed, suggesting that this change might be due to lower statistical power.

**Table 3:** Association analyses of PHQ-D dimensions with gestational age groups and adult socioeconomic and demographic characteristics, with and without adjustment for birth weight percentile as an indicator of fetal growth.

	Model 1 No adjustment for fetal growth		Model 2 Adjusted for fetal growth	
	OR (CI_95_)	p	OR (CI_95_)	p
**Somatoform syndrome**				
GA ≤ 28 weeks	1·38 (0·42, 3·83)	0·56	1·40 (0·43, 3·90)	0·54
GA 29–32 weeks	0·94 (0·35, 2·24)	0·89	0·94 (0·35, 2·24)	0·89
GA 33–36 weeks	0·49 (0·17, 1·20)	0·14	0·51 (0·17, 1·29)	0·17
Gender (female)	5·87 (2·54, 16·07)	<0·001	5·88 (2·55, 16·07)	<0·001
Socioeconomic status	0·87 (0·78, 0·97)	0·02	0·87 (0·78, 0·97)	0·01
BW percentiles			1·00 (0·99, 1·01)	0·74
**Major depressive disorder**				
GA ≤ 28 weeks	**4·06 (1·42, 11·40)**	**0·01**	**4·14 (1·43, 11·77)**	**0·01**
GA 29–32 weeks	0·78 (0·17, 2·63)	0·71	0·78 (0·17, 2·65)	0·72
GA 33–36 weeks	0·75 (0·20, 2·31)	0·63	0·77 (0·20, 2·52)	0·69
Gender (female)	2·02 (0·85, 5·24)	0·12	2·01 (0·85, 5·22)	0·13
Socioeconomic status	0·84 (0·73, 0·96)	0·01	0·84 (0·73, 096)	0·01
BW percentiles			1·00 (0·99, 1·02)	0·82
**Other depressive symptoms**				
GA ≤ 28 weeks	2·07 (0·91, 4·47)	0·07	2·09 (0·91, 4·52)	0·07
GA 29–32 weeks	1·30 (0·67, 2·45)	0·42	1·31 (0·67, 2·46)	0·42
GA 33–36 weeks	1·32 (0·72, 2·35)	0·36	1·33 (0·72, 2·45)	0·36
Gender (female)	1·79 (1·11, 2·96)	0·02	1·79 (1·11, 2·96)	0·02
Socioeconomic status	0·93 (0·86, 1·00)	0·04	0·93 (0·86, 1·00)	0·05
BW percentile			1·00 (0·99, 1·01)	0·88
**Generalized anxiety disorder**				
GA ≤ 28 weeks	**5·27 (1·55, 17·64)**	**0·01**	**5·17 (1·51, 17·37)**	**0·01**
GA 29–32 weeks	2·04 (0·59, 6·60)	0·24	2·03 (0·58, 6·59)	0·24
GA 33–36 weeks	0·84 (0·18, 3·12)	0·81	0·79 (0·16, 3·04)	0·75
Gender (female)	3·86 (1·37, 13·73)	0·02	3·89 (1·39, 13·91)	0·02
Socioeconomic status	0·95 (0·82, 1·11)	0·52	0·94 (0·82, 1·10)	0·49
BW percentile			1·00 (0·98, 1·01)	0·69
**Alcohol-related disorders**				
GA ≤ 28 weeks	**0·13 (0·01, 0·64)**	**0·05**	0·14 (0·01, 0·72)	0·06
GA 29–32 weeks	0·48 (0·17, 1·13)	0·12	0·52 (0·18, 1·24)	0·17
GA 33–36 weeks	0·52 (0·21, 1·19)	0·15	0·63 (0·23, 1·52)	0·32
Gender (female)	0·22 (0·10, 0·45)	<0·001	0·23 (0·10, 0·46)	<0·001
Socioeconomic status	0·92 (0·83, 1·01)	0·10	0·92 (0·83, 1·02)	0·11
BW percentile			1·01 (1·00, 1·02)	0·17

Models 2 was adjusted for birth weight percentiles.

Note: Adjustment for fetal growth did not alter the results, indicating that fetal growth had no significant impact on mental health outcomes.

GA–gestational age; BW Percentile–Birth weight percentile.

Bold text indicates statistically significant associations (p < 0·05) between gestational age categories and the respective mental health outcomes.

## Discussion

This study examined the complex associations between premature birth, birth weight percentiles as a surrogate marker for fetal growth restriction, and the prevalence of various mental health outcomes in adulthood. Our findings demonstrate that prematurity, particularly a gestational age of ≤28 weeks, was associated with significantly higher odds of major depressive disorder and generalized anxiety disorder in adulthood. Each week decrease in gestational age increased the risk for generalized anxiety disorders by 11% and for major depressive disorder by 10%, highlighting a dose-dependent relationship between prematurity and these conditions. Importantly, fetal growth restriction, as indicated by low birth weight percentiles, was not independently associated with mental health outcomes. This distinction suggests that previous studies linking low birth weight to mental health issues may have conflated its effects with those of prematurity. In a sensitivity analysis adjusting for maternal alcohol consumption during pregnancy, the association between extreme prematurity and major depressive disorder was no longer significant, suggesting that prenatal alcohol exposure may mediate this relationship. This finding aligns with prior evidence linking maternal alcohol consumption to an increased risk of depression in offspring. However, the persistence of the association between extreme prematurity and generalized anxiety disorder underscores the unique contribution of gestational age to certain mental health outcomes.

In our cohort, the extremely preterm group has the highest proportion of individuals with low SES, aligning with previously reported socioeconomic challenges faced by preterm individuals.^53^^,^^54^ Several studies have shown that low SES is associated with a higher risk for mental health disorders due to chronic stress exposure, limited access to healthcare, reduced educational opportunities, and fewer social resources.^55^^,^^56^ These factors can contribute to increased psychological vulnerability and hinder coping mechanisms, making individuals with low SES more susceptible to depression and anxiety. Our analyses indicate that higher SES is associated with a reduced risk of somatoform syndrome and major depressive disorder in adulthood. This suggests that socioeconomic resources may serve as a protective factor. When including SES in the multivariable model, the association between the extremely preterm group and an elevated risk of mental health issues remains significant. These findings suggest that the impact of prematurity on mental health is not solely attributable to socioeconomic factors.

Research showed an increased risk of depression in children born preterm. A Swedish study of 86 preterm infants born before 26 weeks gestation from the 1990–1992 cohort found higher rates of mental health issues, such as depression and anxiety, in 11-year-olds (adjusted OR = 2·56, p = 0·036) compared to the control group.^57^ Another Swedish study of 1,301,522 16-year-olds found that those born at 32–36 weeks gestation and before 32 weeks were 1·3 times and 2·9 times more likely respectively to suffer from depressive disorders.^58^ However, this association between preterm birth and depression has not been confirmed in other studies.^59^ In adulthood, only a few studies so far have investigated the link between prematurity and depression. A prospective cohort study of 86,925 postmenopausal women in the Women's Health Initiative found an association between preterm birth (≥4 weeks premature) and depression in adulthood (adjusted OR of 1·18), and lower birth weight was also associated with depression (adjusted OR = 1·21).^60^ Jaekel et al. investigated a cohort of 200 adults born preterm with very low birth weight, providing evidence of an increased risk for mood disorders in adulthood. However, after correcting for multiple testing, the association was not evident.^61^ Boyle et al. examined the prevalence of developmental disabilities in U.S. children and highlighted an overall increase in developmental and behavioral problems. While this study did not directly analyze the impact of preterm birth, it suggests that the improved survival rates of preterm infants may contribute to the rising prevalence of these challenges.^62^ A 2014 study found that preterm birth was linked to a significantly increased risk of affective disorders like depression, even after adjusting for birth weight.^63^ Conversely, the Helsinki Birth Cohort Study found that while men born late preterm had an increased risk of suicide, late preterm birth did not generally impact adult psychopathology on a broader scale.^64^ Most studies have been retrospective cohort studies linking birth data with ICD codes in national health registries, potentially missing undiagnosed depression. The association between premature birth and depression observed in this study could arise from various factors, and is notably present only in individuals born at or before 28 weeks of gestational age (OR = 4·14, p = 0·01), but not in those born prematurely after this threshold. Additionally, no association was found between birth weight percentile and the presence of major depressive disorder in adulthood, with prematurity being the sole contributing factor. Our findings regarding the association between prematurity and depression in adulthood align with theoretical frameworks such as the DOHaD model. This model posits that early life stressors, including preterm birth, can disrupt neurodevelopment, creating long-term vulnerability to mental disorders. Prenatal programming, a key component of this framework, suggests that adverse intrauterine conditions, such as nutrient deprivation or maternal stress, induce permanent changes in the developing brain and stress-regulatory systems. By integrating these perspectives, our findings contribute to a growing body of evidence that highlights the lasting impact of prematurity on adult mental health. Several studies show that psychosocial stressors during pregnancy increase the risk of premature birth.^65^^,^^66^ Babies born to depressed mothers have significantly lower levels of serotonin and dopamine, and higher levels of cortisol compared to babies born to non-depressed mothers.^63^^,^^67^ Moreover, premature babies often suffer from neurodevelopmental challenges^68^ and behavioral issues^32^ which could impact mental health. Our analyses demonstrated an association between emotional problems, peer problems, and the total difficulties score at ages 2–4 years (as reported by mothers) and major depressive disorder in adulthood but these associations were only present in individuals born preterm. Other studies report that emotional and peer problems are particularly common in children born preterm.^69^^,^^70^ The association between behavioral problems and the development of mental outcomes is posited by the social defeat hypothesis that suggests that the risk of psychiatric issues increases due to the influence of socioemotional difficulties like social defeat in peer groups.^71^^,^^72^ This could provide another possible explanation for the association between gestational age of ≤28 weeks with major depressive disorder in adulthood in our study. This highlights the importance of supporting extremely premature infants and their parents to reduce the risk of major depressive disorder later in life. Interviews with parents of infants born at 24 weeks of gestational age revealed difficulties in managing their children's special needs and a desire for more support from healthcare and schools.^73^ In the sensitivity analysis, which included alcohol consumption during pregnancy, the association between preterm birth and major depressive disorder was no longer observed. This may be attributed to the small number of mothers who smoked or consumed alcohol during pregnancy, resulting in higher statistical model complexity. Alternatively, maternal alcohol consumption during pregnancy may act as a confounding factor, influencing both the likelihood of preterm birth^28^ and the subsequent development of mental health disorders, such as depression, in offspring.^31^ Several studies have demonstrated this association.^31^^,^^74^^,^^75^ For instance, O'Connor et al. reported that children exposed to alcohol prenatally exhibited more depressive symptoms than their non-exposed peers.^74^ Similarly, longitudinal evidence from Easey et al., based on a cohort of 13,480 participants, demonstrated a significant association between prenatal alcohol exposure and depression in young adulthood, with effects persisting into later life.^31^ These findings are complemented by evidence linking maternal smoking during pregnancy to an increased risk of mood and anxiety disorders in offspring.^30^

Our study demonstrated an association between prematurity at or before 28 weeks of gestational age and the higher odds of having generalized anxiety disorder in adulthood (OR = 5·17, p = 0·01). Fetal growth restriction was not a contributing factor, and thus, prematurity alone is associated with generalized anxiety disorder in adulthood. A systematic review and meta-analysis of six studies involving 1519 adolescents found that those born very preterm (<32 weeks gestation) had nearly twice the risk of clinically significant anxiety (odds ratio 2·27) compared to the control group but the study did not differentiate between prematurity and fetal growth.^76^ A recent study by Kong et al.^77^ investigated the associations between preterm birth with a wide spectrum of neurodevelopmental and psychiatric disorders in children and adolescents aged 4–22 years. The authors reported that preterm birth was significantly associated with increased risks for anxiety disorders, intellectual disabilities, specific developmental disorders, autism spectrum disorders, attention-deficit/hyperactivity disorders, and other emotional and behavioral disorders. The association shown could be due to several causes. Firstly, individuals born preterm more often display disrupted brain structures than individuals born at term,^78^^,^^79^ therefore abnormal brain development could contribute to disrupted anxiety learning processes which may then persist into adulthood.^80^ Schmitz-Koep et al. investigated MRI volumes of 101 preterm infants (<32 weeks of gestational age and/or birth weight below 1500 g) and 108 controls at the age of 26 years, showing significantly lower whole amygdala volumes in the individuals born preterm. However, this anatomical change showed no association with avoidant personality acting as a surrogate marker for social anxiety traits.^81^ In addition to brain development, post-birth conditions may be crucial. A rat study found that early pain experiences, similar to those of premature infants in neonatal intensive care, can disrupt fear memory by impairing hippocampal neural circuit development.^82^ The increased risk of anxiety disorder and behavioral problems and thus possible isolation in peer circles probably also play a role in premature infants.^83^ This finding highlights the need for early psychological screening and intervention for extremely preterm infants to reduce these risks.^55^^,^^56^

Our findings highlight the significant impact of preterm birth (gestational age of ≤28 weeks) on mental health outcomes in adulthood but there was no association between birth weight percentile and mental health outcomes in adulthood. This suggests that, beyond nutritional status, a complex interplay between abnormal brain development and postnatal environmental factors likely contributes to the development of mental health disorders in preterm infants. Thus, the nutritional state may not be as important as abnormal brain development and postnatal conditions in the development of mental health outcomes in preterm infants. The findings align with the DOHaD theory, which suggests that early stressors, such as premature birth, can disrupt neurodevelopment and thereby create a long-term vulnerability to mental disorders. Our study highlights that both neurobiological and environmental challenges inherent to preterm birth jointly influence mental health outcomes later in life. More investigation is required to disentangle how incomplete brain development and postnatal environmental exposures interact to elevate mental health risks. Further research is needed to differentiate whether the heightened risk is primarily due to incomplete brain development during premature birth or if it stems more from environmental factors encountered after birth. It is important to interpret the study findings with caution, as the limited screening timeframe may affect the generalizability of our conclusions. Replication of these findings in larger and longitudinal cohorts is essential to deepen our understanding of the multifactorial relationship between preterm birth and mental health outcomes in adulthood.

This study has several limitations. Firstly, the findings may not be generalizable as this was a single-center, hospital-based cohort study. Difficulties in contacting participants and some opting out may lead to selection bias. Most participants were of white ethnicity limiting the applicability of the conclusions to this group. The exploratory nature of the study means no corrections for multiple testing were made. Reliance on self-report questionnaires for mental outcomes and retrospective reporting of behavioral difficulties by mothers could introduce recall bias. One potential consideration in our study is the reliance on a subsample of 217 participants (35.8% of the total sample) for whom maternal SDQ reports were available. While this response rate may appear limited, it is consistent with similar studies involving retrospective maternal reports and long follow-up intervals. For instance, studies on prenatal stress and childhood outcomes often report response rates of approximately 30–40%, reflecting the challenges of collecting retrospective data decades after the initial events. Importantly, the absolute size of our subsample (N = 217) remains one of the largest to date in the field of preterm birth research investigating early childhood behavioral characteristics and adult mental health outcomes. This highlights the relevance and potential generalizability of our findings, even within the constraints of retrospective data collection. Nevertheless, we acknowledge the potential for selection bias and the limitations inherent in relying on retrospective maternal recall, which may be subject to inaccuracies due to the time elapsed. Future studies with prospectively collected data could provide additional insights into these developmental pathways. Moreover, while structured clinical interviews are the gold standard for diagnosing mental disorders, they were not used due to their burden on participants, opting instead for personal medical history interviews to clarify ambiguities. Additionally, it was not possible to analyze longitudinal traits, as this was a cross-sectional study, which limits the ability to observe changes in mental health outcomes over time. Another limitation of this study concerns the assessment of mental health, which was based on current symptomatology. The instruments employed, the PHQ-D and GAD-7, assess depressive and anxiety symptoms within relatively short time frames, typically covering the past two to four weeks. Although these tools are well-validated and frequently used in epidemiological research, their limited temporal scope may not capture chronic or episodic mental disorders comprehensively. Depressive and anxiety symptoms can fluctuate, and participants who were symptom-free during the assessment window may have experienced significant psychiatric symptoms before or after this period. As such, our findings may underestimate the true prevalence of persistent or episodic mental disorders in this cohort. At the same time, the limited 2–4 week timeframe of the questionnaires means that our findings could also potentially overestimate the occurrence of mental health symptoms among participants. Future studies should consider using assessment tools that capture longer-term mental health trends to provide a more thorough understanding of participants' psychiatric histories. In addition, another limitation is that medical care for preterm infants changed substantially during the study recruitment period, which may affect the generalizability of results across participants born in different periods of time. Advances in standards of care and treatment of newborns during this period, such as improved survival rates for preterm infants, could lead to differences in long-term outcomes between older and younger participants. In addition, methods for estimating gestational age have evolved, using maternal reporting in the early years and ultrasound in later years, leading to greater precision in more recent cohorts. These changes could affect the comparability of gestational age across birth years, but we do not believe that they have significantly distorted the conclusions of the study, as we adjusted for age in our analyses to account for possible generational differences. One potential limitation of our study is the exclusion of neonatal acuity measures, such as length of NICU stay, from the final analysis. Although gestational age and birthweight are commonly used predictors in studies of preterm birth outcomes, neonatal acuity factors, such as NICU stay duration, might provide additional insights into the variability of adult mental health outcomes. To address this, we conducted a sensitivity analysis that incorporated NICU stay duration as an indicator of neonatal health severity. While we observed substantial variability in NICU stay durations across groups (e.g., 72·7 days, SD = 32·3, for Group 4 vs. 5·3 days, SD = 13·3, for Group 2), no significant associations with major depressive disorder (OR = 1·01, p = 0·57) or anxiety disorders (OR = 1.02, p = 0·07) were found. Given these findings, and to maintain model simplicity, we chose not to include this variable in our final analysis. However, future research may benefit from further exploration of neonatal acuity measures and their potential interaction with other predictors, such as gestational age and birth weight. Despite its limitations, the current study examined an extraordinarily large cohort of adults born preterm, with varying degrees of prematurity, alongside a substantial control group. A thorough assessment of perinatal medical history was also performed through the review of medical charts. Another strength of this study is the use of external anamnesis, where mothers of participants provided detailed questionnaires on childhood emotional problems, adding a valuable perspective to our findings.

A gestational age of ≤28 weeks was associated with a higher prevalence of major depressive disorder and generalized anxiety disorder in adulthood but not somatoform syndrome or other depressive symptoms. The association between major depressive disorder and prematurity may be explained by higher rates of maternal alcohol consumption during pregnancy among preterm infants, while the association between prematurity and generalized anxiety disorder remains significant independent of this factor. Notably, fetal growth restriction, indicated by birth weight percentiles, does not seem to influence later mental health, suggesting that previous analyses linking low birth weight to mental health issues were likely confounded by the high correlation between prematurity and low birth weight, rather than a direct effect of hypotrophy. Overall, these findings highlight a possible vulnerability of the extremely preterm group to mental health issues in adulthood. Consistent with the DOHaD theory, our findings support the idea that early life factors, such as extreme prematurity, may contribute to an increased risk of mental health disorders in adulthood.

## Contributors

AF and AKS conceived and designed the study. AH performed the statistical analysis. AF, AH, SG, and JT drafted the initial manuscript. AF and AH accessed and verified the underlying data. All authors contributed to data interpretation, reviewed the manuscript, approved the submitted version, had full access to all the data reported in the study, and had final responsibility for the decision to submit for publication.

## Data sharing statement

Data are available upon reasonable request. Access to data, responsibility, and analysis: The analysis presents the clinical data of a cohort. This project constitutes a major scientific effort with high methodological standards and detailed guidelines for analysis and publication to ensure scientific analyses are on the highest level, therefore, data are not made available for the scientific community outside the established and controlled workflows and algorithms. To meet the general idea of verification and reproducibility of scientific findings, we offer access to data at the local database upon request at any time. Interested researchers should make their requests to the coordinating PI of the GPS (Achim Fieβ; achim.fiess@unimedizin-mainz.de). More detailed contact information is available at the homepages of the UM (www.unimedizin-mainz.de).

## Declaration of interests

Funding/support All authors: The Gutenberg Prematurity Study was supported by the Ernst und Berta-Grimmke Stiftung, Stufe 1 support of the UM and the Else Kröner-Fresenius-Stiftung. The funders had no role in study design, data collection and analysis, decision to publish, or preparation of the manuscript. Schuster AK holds the professorship for ophthalmic healthcare research endowed by “Stiftung Auge” and financed by “Deutsche Ophthalmologische Gesellschaft” and “Berufsverband der Augenärzte Deutschlands e.V.”. Tesarz J. serves as a member of the Council of Scholars of the EMDRIA Global Alliance and as mandate holder for the German clinical practice guidelines on multimorbidity, irritable bowel syndrome, and neuroborreliosis on behalf of the German College of Psychosomatic Medicine (Deutsches Kollegium für Psychosomatische Medizin, DKPM) and the German Society of Psychosomatic Medicine and Psychotherapy (Deutsche Gesellschaft für Psychosomatische Medizin und Ärztliche Psychotherapie, DGPM). Fieβ A, Hartmann A, Ernst M, Mildenberger E, Brähler E, Urschitz MS, Pfeiffer N, Beutel ME, and Giβler S: none.

Financial disclosure/conflict of interest: Schuster AK receives research support from Santen, Abbvie, Norvartis, and Heidelberg Engineering. Tesarz J receives research support from the Bundesministerium für Bildung und Forschung (BMBF – Federal Ministry of Education and Research, Germany) and the Deutsche Forschungsgemeinschaft (DFG – German Research Foundation). Fieβ A, Hartmann A, Ernst M, Mildenberger E, Brähler E, Urschitz MS, Pfeiffer N, Beutel ME, and Giβler S: none.
