# Supplementary material for: Sequelae of preterm birth over the lifespan: an exploratory analysis of behavioral problems in childhood and increased risk of major depression and anxiety in adulthood from a cohort study
Source: eClinicalMedicine. 2025 Jul 8;85:103316. doi: 10.1016/j.eclinm.2025.103316 (PMC12274871; doi:10.1016/j.eclinm.2025.103316)
Supplement: Supplemental Figure and Tables [file mmc1.pdf]

Supplementary Materials

Supplementary Figure 1 – Flow chart of study inclusion and exlusion criteria

Supplementary Table 1 - Association analyses of PHQ-D dimensions with peri- and postnatal parameters with and without adjustment for fetal growth (employing continuous parameters)

Supplementary Table 2 - Sensitivity analysis of PHQ-D dimensions with peri- and postnatal parameters with additional integration of maternal smoking and alcohol consumption during pregnancy

Supplementary Table 3 - Sensitivity analysis of PHQ-D dimensions with peri- and postnatal parameters with additional integration of examination before pandemic begin/after pandemic begin (2020-03-11)

Supplementary Table 4 - Association analyses between mental disorders in adulthood with strength and difficulties in childhood (2-4 years of age) as retrospectively reported by mothers several years later.

Suppl. Figure 1. Flow chart of study inclusion and exlusion criteria.

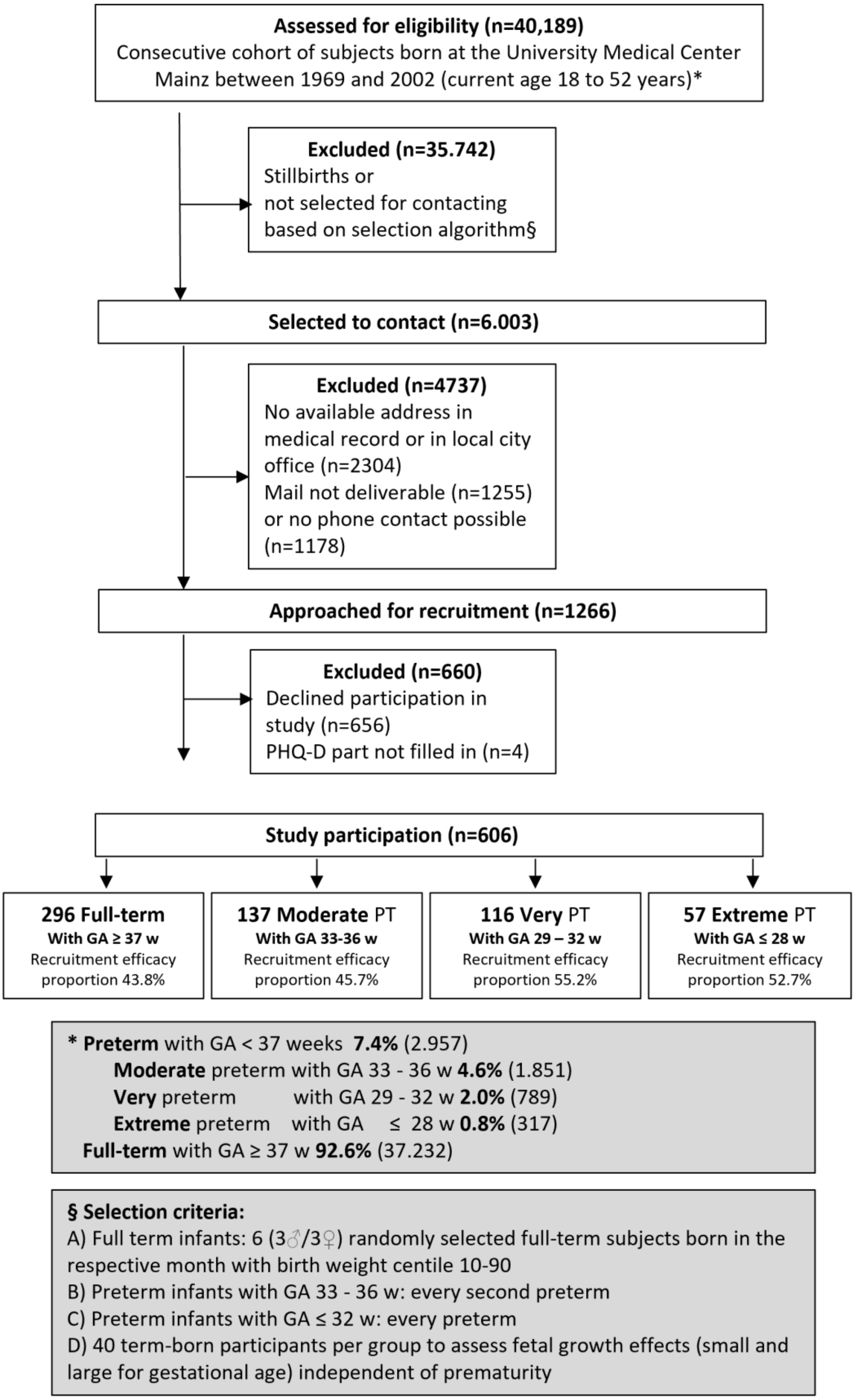

Legend: PT- preterm; GA – gestational age

Suppl. Table 1 Association analyses of PHQ-D dimensions with peri- and postnatal parameters with and without adjustment for fetal growth (employing continuous parameters).

	**Model 1**<br>**No adjustment for fetal growth**		**Model 2**<br>**Adjusted for fetal growth**	
	OR (CI_{95})	*p*	OR (CI_{95})	*p*
**Somatoform syndrome**				
Gestational age (weeks)	0·99<br>(0·92, 1·07)	0·83	0·99<br>(0·92, 1·07)	0·73
BW percentiles			1·00<br>(0·99, 1·01)	0·44
**Major depressive syndrome**				
Gestational age (weeks)	0·91<br>(0·83, 0·99)	0·02	0·90<br>(0·83, 0·98)	0·02
BW percentiles			1·00<br>(0·99, 1·02)	0·58
**Other depressive symptoms**				
Gestational age (weeks)	0·96<br>(0·91, 1·01)	0·14	0·96<br>(0·91, 1·01)	0·14
BW Percentile			1·00<br>(0·99, 1·01)	0·89
**Anxiety disorders**				
Gestational age (weeks)	0·89<br>(0·80, 0·97)	0·01	0·89<br>(0·80, 0·97)	0·01
BW Percentile			1·00<br>(0·98, 1·01)	0·99
**Alcohol-related disorders**				
Gestational age (weeks)	1·12<br>(1·05, 1·23)	0·003	1·11<br>(1·03, 1·22)	0·01
BW Percentile			1·01<br>(1·00, 1·01)	0·31

Legend: Model 1 and model 2 were adjusted for socioeconomic status, gender, and age, with birth weight percentile integrated as an additional parameter in model 2. Note: Adjustment for fetal growth did not alter the results, indicating that fetal growth had no significant impact on mental health outcomes.

BW Percentile – Birth weight percentile

Suppl. Table 2. Sensitivity analysis of PHQ-D dimensions with peri- and postnatal parameters with additional integration of maternal smoking and alcohol consumption during pregnancy.

	**Multivariable model adjusted for age**	
	OR (CI_{95})	p
**Somatoform syndrome**		
GA≤28 weeks	1·05 (0·28, 3·19)	0·93
GA 29–32 weeks	0·91 (0·33, 2·22)	0·84
GA 33-36 weeks	0·56 (0·19, 1·44)	0·25
Gender (female)	6·09 (2·58, 17·10)	<0·001
Socioeconomic status	0·88 (0·79, 0·98)	0·03
BW Percentile	1·00 (0·99, 1·01)	0·56
Maternal smoking during pregnancy (yes)	0·60 (0·11, 2·18)	0·49
Maternal alcohol consumption during pregnancy (yes)	9·60 (2·69, 32·86)	<0·001
**Major depressive disorder**		
GA≤28 weeks	2·84 (0·85, 9·07)	0·08
GA 29–32 weeks	0·66 (0·13, 2·41)	0·56
GA 33-36 weeks	0·86 (0·21, 2·99)	0·82
Gender (female)	1·86 (0·74, 5·09)	0·20
Socioeconomic status	0·87 (0·75, 1·01)	0·07
BW Percentile	1·00 (0·99, 1·02)	0·55
Maternal smoking during pregnancy (yes)	1·57 (0·36, 5·33)	0·51
Maternal alcohol consumption during pregnancy (yes)	18·60 (5·24, 65·76)	<0·001
**Other depressive symptoms**		

GA≤28 weeks	2·27<br>(0·98, 4·96)	0·05
GA 29–32 weeks	1·32<br>(0·67, 2·48)	0·41
GA 33-36 weeks	1·32<br>(0·71, 2·42)	0·37
Gender (female)	1·81<br>(1·11, 2·99)	0·02
Socioeconomic status	0·92<br>(0·85, 0·99)	0·03
BW Percentile	1·00<br>(0·99, 1·01)	0·94
Maternal smoking during pregnancy (yes)	0·97<br>(0·35, 2·33)	0·94
Maternal alcohol consumption during pregnancy (yes)	0·26<br>(0·01, 1·38)	0·20
**Generalized anxiety disorder**		
GA≤28 weeks	4·63<br>(1·27, 16·22)	0·02
GA 29–32 weeks	2·05<br>(0·58, 6·71)	0·24
GA 33-36 weeks	0·88<br>(0·18, 3·45)	0·86
Gender (female)	3·89<br>(1·37, 14·07)	0·02
Socioeconomic status	0·95<br>(0·82, 1·11)	0·54
Maternal smoking during pregnancy (yes)	0·42<br>(0·02, 2·32)	0·42
Maternal alcohol consumption during pregnancy (yes)	4·64<br>(0·87, 19·29)	0·05

The multivariable analysis was adjusted for age· The dependent variable 'Alcohol-related disorders' was excluded from this analysis, as none of the individuals with alcohol problems had mothers who smoked or consumed alcohol during pregnancy, making it impossible to estimate effects for this group.

GA – gestational age; BW Percentile – Birth weight percentile

Suppl. Table 3. Sensitivity analysis of PHQ-D dimensions with peri- and postnatal parameters with additional integration of examination before pandemic begin/after pandemic begin (2020-03-11).

	**Multivariable model adjusted for age**	
	OR (CI_{95})	p
**Somatoform syndrome**		
GA≤28 weeks	1·58 (0·45, 4·90)	0·45
GA 29–32 weeks	0·99 (0·37, 2·43)	0·99
GA 33-36 weeks	0·52 (0·18, 1·32)	0·19
Gender (female)	5·93 (2·56, 16·23)	<0·001
Socioeconomic status	0·87 (0·78, 0·97)	0·01
BW Percentile	1·00 (0·99, 1·01)	0·74
Pre-pandemic (yes)	0·82 (0·38, 1·71)	0·60
**Major depressive disorder**		
GA≤28 weeks	3·90 (1·20, 12·81)	0·02
GA 29–32 weeks	0·77 (0·16, 2·64)	0·70
GA 33-36 weeks	0·77 (0·20, 2·51)	0·68
Gender (female)	2·01 (0·85, 5·20)	0·13
Socioeconomic status	0·84 (0·73, 0·97)	0·02
BW Percentile	1·001 (0·99, 1·02)	0·81
Pre-pandemic (yes)	1·11 (0·42, 2·84)	0·83
**Other depressive symptoms**		
GA≤28 weeks	2·51 (1·04, 5·82)	0·03
GA 29–32 weeks	1·40 (0·71, 2·67)	0·31
GA 33-36 weeks	1·39 ((0·74, 2·56)	0·29
Gender (female)	1·81 (1·11, 2·99)	0·02

Socioeconomic status	0·92<br>(0·86, 0·99)	0·04
BW Percentile	1·00<br>(0·99, 1·01)	0·89
Pre-pandemic (yes)	0·73<br>(0·43, 1·23)	0·24
**Generalized anxiety disorder**		
GA≤28 weeks	3·52<br>(0·94, 13·28)	0·06
GA 29–32 weeks	1·67<br>(0·47, 5·61)	0·41
GA 33-36 weeks	0·72<br>(0·15, 2·83)	0·66
Gender (female)	3·77<br>(1·35, 13·45)	0·02
Socioeconomic status	0·96<br>(0·82, 1·12)	0·61
BW Percentile	1·00<br>(0·98, 1·01)	0·74
Pre-pandemic (yes)	2·01<br>(0·72, 5·89)	0·19

The multivariable analysis was adjusted for age. The dependent variable 'Alcohol-related disorders' was excluded from this analysis, as none of the individuals with alcohol problems had mothers who smoked or consumed alcohol during pregnancy, making it impossible to estimate effects for this group· Reference for pre-pandemic comparison: after pandemic began.

GA – gestational age; BW Percentile – Birth weight percentile

Suppl. Table 4 Association analyses between mental disorders in adulthood with strength and difficulties in childhood (2-4 years of age) as retrospectively reported by mothers several years later.

	**Univariable Model, adjusted for age, gender and socioeconomic-status**			
	Preterm		Term	
	OR (CI_{95})	*p*	OR (CI_{95})	*p*
**Somatoform syndrome**				
Conduct problems, age 2-4 years	1·85 (0·84, 4·81)	0·14	0·88 (0·37, 1·77)	0·75
Emotional problems, age 2-4 years	0·98 (0·48, 1·80)	0·95	0·75 (0·36, 1·26)	0·35
Hyperactivity, age 2-4 years	0·75 (0·36, 1·23)	0·34	1·13 (0·78, 1·60)	0·49
Peer problems, age 2-4 years	1·06 (0·62, 1·66)	0·80	0·99 (0·49, 1·77)	0·98
Prosocial score, age 2-4 years	0·91 (0·56, 1·49)	0·69	1·03 (0·56, 1·49)	0·87
Total difficulties score, age 2-4 years	1·01 (0·81, 1·24)	0·96	0·98 (0·77, 1·24)	0·88
**Major depressive syndrome**				
Conduct problems, age 2-4 years	1·22 (0·68, 2·02)	0·45	0·87 (0·33, 1·77)	0·73
Emotional problems, age 2-4 years	1·42 (1·00, 2·05)	0·05	1·08 (0·61, 1·72)	0·75
Hyperactivity, age 2-4 years	1·04 (0·76, 1·40)	0·78	1·12 (0·71, 1·67)	0·61
Peer problems, age 2-4 years	1·71 (1·26, 2·44)	0·001	0·29 (0·02, 1·05)	0·20
Prosocial score, age 2-4 years	0·92 (0·63, 1·39)	0·65	1·43 (0·85, 2·99)	0·25
Total difficulties score, age 2-4 years	1·18 (1·03, 1·36)	0·02	0·94 (0·71, 1·19)	0·63
**Other depressive symptoms**				
Conduct problems, age 2-4 years	0·91 (0·57, 1·36)	0·67	0·50 (0·21, 1·00)	0·08
Emotional problems, age 2-4 years	1·22 (0·94, 1·58)	0·12	1·10 (0·77, 1·53)	0·57
Hyperactivity, age 2-4 years	0·84 (0·66, 1·05)	0·16	0·96 (0·71, 1·26)	0·78

Peer problems, age 2-4 years	1·11<br>(0·85, 1·42)	0·40	1·07<br>(0·64, 1·66)	0·77
Prosocial score, age 2-4 years	1·00<br>(0·77, 1·33)	0·98	1·23<br>(0·89, 1·84)	0·24
Total difficulties score, age 2-4 years	1·01<br>(0·90, 1·13)	0·79	0·91<br>(0·74, 1·10)	0·36
**Anxiety disorder**				
Conduct problems, age 2-4 years	1·20<br>(0·70, 1·92)	0·46	1·37<br>(0·63, 2·75)	0·37
Emotional problems, age 2-4 years	1·12<br>(0·73, 1·64)	0·57	0·87<br>(0·43, 1·42)	0·63
Hyperactivity, age 2-4 years	0·99<br>(0·73, 1·28)	0·92	1·39<br>(0·95, 2·06)	0·08
Peer problems, age 2-4 years	1·13<br>(0·80, 1·51)	0·43	0·75<br>(0·26, 1·53)	0·51
Prosocial score, age 2-4 years	0·96<br>(0·68, 1·40)	0·80	1·25<br>(0·80, 2·26)	0·39
Total difficulties score, age 2-4 years	1·06<br>(0·91, 1·21)	0·44	1·05<br>(0·80, 1·34)	0·72
**Alcohol-related disorders**				
Conduct problems, age 2-4 years	0·85<br>(0·26, 1·88)	0·73	1·20<br>(0·58, 2·26)	0·59
Emotional problems, age 2-4 years	0·95<br>(0·48, 1·55)	0·86	0·58<br>(0·23, 1·06)	0·15
Hyperactivity, age 2-4 years	0·80<br>(0·40, 1·29)	0·43	0·83<br>(0·51, 1·21)	0·39
Peer problems, age 2-4 years	0·74<br>(0·20, 1·47)	0·54	0·63<br>(0·23, 1·17)	0·23
Prosocial score, age 2-4 years	1·00<br>(0·59, 1·96)	0·99	1·57<br>(1·03, 2·73)	0·06
Total difficulties score, age 2-4 years	0·94<br>(0·66, 1·19)	0·67	0·90<br>(0·67, 1·16)	0·45
